# Ablating *Satb1* reprograms the differentiation trajectory of exhausted CD8^+^ T subsets to enhance antitumor immunity

**DOI:** 10.3389/fimmu.2026.1744549

**Published:** 2026-06-22

**Authors:** Linwei Wu, Haolin Jiang, Jianling Gao, Xiaolan Ji, Yu Chen, Zhenghao Ma, Xinying Li, Jin Wang, Yachen Zang, Ji Zhang, Xin Zhao, Xuefeng Wang

**Affiliations:** 1 The First Affiliated Hospital of Soochow University and The Second Affiliated Hospital of Soochow University and The Fourth Affiliated Hospital of Soochow University and School of Basic Medical Sciences, Suzhou Medical College, Soochow University, Suzhou, China; 2 Department of General Surgery, The First Affiliated Hospital of Soochow University, Suzhou, China; 3 Department of Critical Care Medicine, The Fourth Affiliated Hospital of Soochow University, Suzhou, China; 4 Department of Ophthalmology, The Second Affiliated Hospital of Soochow University, Suzhou, China; 5 Department of General Surgery, The Fourth Affiliated Hospital of Soochow University, (Suzhou Dushu Lake Hospital), Suzhou, China; 6 Department of Urology, The Second Affiliated Hospital of Soochow University, Suzhou, China

**Keywords:** effector-like Tex- int cells, Satb1, stem-like Tpex cells, Tex-int cells, Tpex cells, tumor immunity, tumor-infiltrating CD8+ T cells

## Abstract

**Introduction:**

Under chronic infections or in tumors, persistent antigen exposure drives CD8^+^ T cell exhaustion, a heterogeneous state encompassing a differentiation continuum from stem-like progenitor (Tpex) cells through transitory effector-like (Tex-int) cells to terminally exhausted (Tex-term) subsets. Among these T cell subsets, Tex-int cells serve as the primary population responsible for direct tumor cell killing. However, the intrinsic regulatory mechanisms that govern the Tpex-to-Tex-int transition remain incompletely defined.

**Methods:**

In this study, we explore the role of special AT-rich sequence-binding protein 1 (SATB1) in the differentiation of Tex-int cells from their precursors. We observed downregulation of SATB1 during Tpex-to-Tex-int differentiation in tumors. Notably, the genetic ablation of *Satb1* in T cells markedly expanded the population of tumor-infiltrating CD8^+^ T cells (CD8^+^ TILs).

**Results:**

Ablating *Satb1* not only promoted the differentiation of Tex-int cells from Tpex cells within the tumor microenvironment but also remodeled T cell differentiation in tumor-draining lymph nodes (TdLNs) by expanding the Tpex pool from tumor-specific memory CD8^+^ T cells (T_TSM_) and driving the Tpex1 to Tpex2 transition, thereby augmenting Tex-int production in tumors. Although early-stage Tex-int cells in *Satb1*-deficient mice displayed transient functional impairment relative to controls, this difference was no longer evident in late-stage tumors, where sustained Tex-int accumulation correlated with significantly suppressed tumor growth and prolonged survival.

**Discussion:**

Our results identify SATB1 as a pivotal regulator of exhausted CD8^+^ T cell subset differentiation and suggest its targeting as a promising strategy to expand the Tex-int population for enhanced cancer immunotherapy.

## 1 Introduction

During acute infections, effector CD8^+^ T cells differentiate into short-lived effector cells (SLECs) or memory precursor effector cells (MPECs), with the latter giving rise to memory CD8^+^ T cells following antigen clearance (1–3). Conversely, chronic infections and tumors drive CD8^+^ T cells into a state of exhaustion due to persistent antigen exposure (4–6). These exhausted T cells upregulate inhibitory receptors such as programmed cell death protein-1 (PD-1) and T-cell immunoglobulin and ITIM domain protein (TIM-3) and exhibit functional impairments distinct from their effector or memory counterparts (7–9).

Accumulating data have delineated a cellular hierarchy within the heterogeneous exhausted CD8^+^ T cell pool, comprising precursor of exhausted T (Tpex1) cells, progenitor of exhausted T (Tpex2) cells, effector-like exhausted T (Tex-int) cells and terminally exhausted T (Tex-term) cells (10–12). The first two subsets, collectively termed stem-like CD8^+^ T cells, are governed by the transcription factor TCF-1 (13). These Tpex cells not only possess self-renewal capacity and serve as the primary responders to immune checkpoint blockade (ICB) but also continuously differentiate into downstream Tex subsets to sustain the exhausted T cell pool and mediate tumor control (14–16). During tumor progression, Tpex cells are constitutively recruited from peripheral lymphoid organs into tumors to replenish the intratumoral T cell compartment (17). Furthermore, a fraction of these cells traffics back to tumor-draining lymph nodes (TdLNs) via lymphatic vessels, thereby maintaining a peripheral reservoir to support durable immune responses (18).

Positioned along the differentiation trajectory from Tpex to terminal exhaustion, Tex-int subset represents a critical intermediate. These cells, characterized by CX3CR1 expression, maintain proliferative and effector functions despite displaying exhaustion markers, which establishes them as the principal cytotoxic population among exhausted T cells (19, 20). They exhibit higher surface levels of PD-1 and TIM-3 than Tpex cells but possess more limited self-renewal capacity, instead displaying heightened effector activity (21–23). As Tex-int cells differentiate into Tex-term cells, they undergo a progressive decline in both proliferative potential and functional capacity. Thus, the formation of Tex-int cells is indispensable for durable antitumor immunity, making the investigation of their differentiation from Tpex precursors essential for understanding immune evasion and informing next-generation immunotherapies (24).

As the central inductive site for anti-tumor T cell immunity, TdLNs harbors T_TSM_ population that function as upstream precursors to Tpex cells. This subset maintains an epigenetic state unmarked by TOX-driven exhaustion programming, characterized by a TCF-1^+^TOX^-^ phenotype and endowed with self-renewal capacity and substantial proliferative potential (10, 25, 26). Following chronic antigen stimulation, T_TSM_ commits to the CD62L^+^Tpex and CD62L^-^Tpex lineages, concurrently initiating a migration program toward tumor tissues where they ultimately differentiate into effector Tex-int cells to eliminate malignant cells (18, 27, 28).

Emerging evidence implicates SATB1 as a key regulator of CD8^+^ T cell biology in tumors (29–31). In the tumor microenvironment, TGF-β signaling suppresses SATB1 expression via SMAD protein recruitment to the Satb1 promoter, a mechanism that contributes to enhanced PD-1 expression (32). scRNA-seq profiling has identified CD62L^+^Tpex1 cells as a distinct population with a unique transcriptional profile that serves as the origin for the exhausted T cell fate, giving rise to CD62L^-^Tpex2 and subsequently Tex-int cells (33). SATB1 is critical in this hierarchy, where it helps maintain Tpex1 cell quiescence and likely orchestrates the timing of their differentiation (34, 35).

In the current study, we investigated the role of SATB1 in regulating the expansion of CD8^+^ T cells and the differentiation of Tex-int cells during antitumor immune responses. Our findings provide new theoretical insights for the advancement of cancer immunotherapy.

## 2 Results

### 2.1 SATB1 expression is downregulated in effector-like Tex-int subset compared to Tpex cells in tumors

While stem-like Tpex cells with self-renewal capacity are essential for maintaining CD8^+^ T cell pool in the tumor microenvironment, their ability to efficiently differentiate into effector-like Tex-int cells, the primary cell population responsible for killing tumors, governs antitumor immunity. Consequently, defining the mechanisms underlying Tpex-to-Tex-int differentiation is a critical objective that could lead to novel immunotherapy strategies (36).

**Figure 1:**
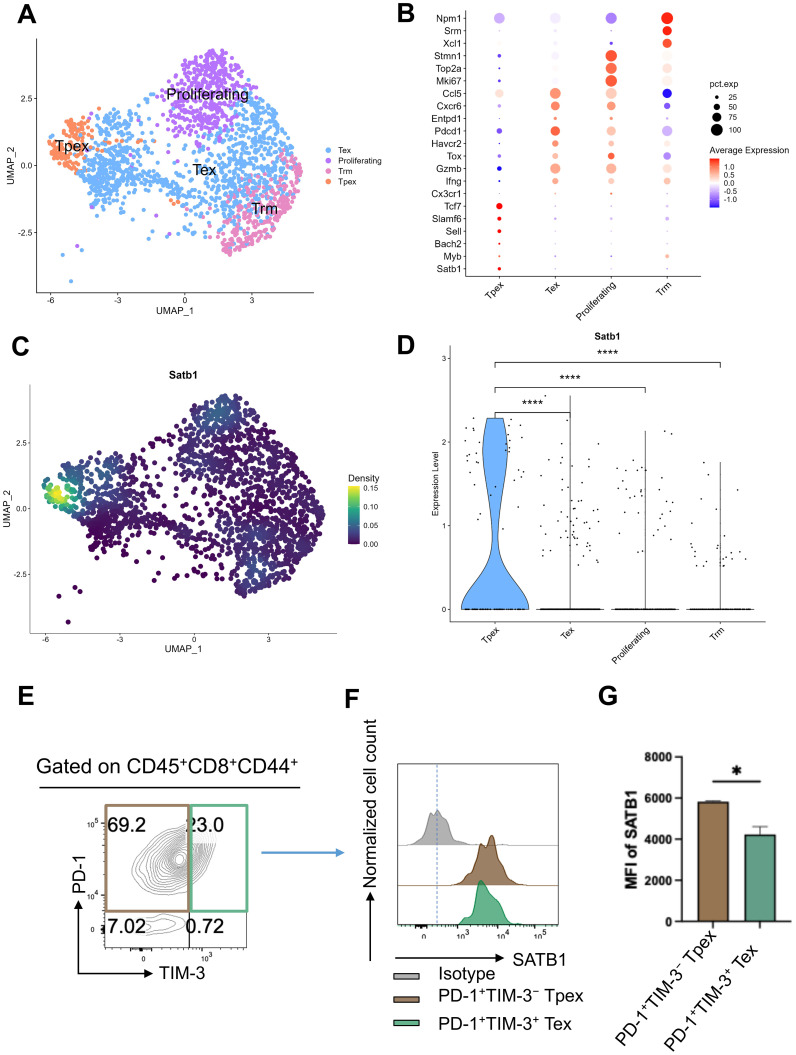
SATB1 expression was downregulated in Tex-int cells compared to Tpex cells in tumors. **(A–D)** scRNA-seq analysis of CD8^+^ TILs from B16 tumor-bearing mice. **(A)** UMAP projection showing the clustering of CD8^+^ TILs into Tpex, Tex, tissue-resident memory T (Trm) and proliferating subsets. **(B)** Dot plot showing the expression of canonical genes across the identified CD8^+^ TIL subsets. **(C)** UMAP feature plots visualizing the expression of *Satb1*. **(D)** Violin plot showing the expression levels of Satb1 across CD8^+^ T cell subsets. **(E–G)** Flow cytometric analysis of SATB1 protein expression in defined CD8^+^ TIL subsets. **(E)** Representative flow cytometry plots gated on CD8^+^ TILs, showing the identification of PD-1^+^TIM-3^-^ Tpex and PD-1^+^TIM-3^+^ Tex cells. **(F)** Representative histograms showing SATB1 expression in the gated PD-1^+^TIM-3^-^ Tpex and PD-1^+^TIM-3^+^ Tex cell populations. **(G)** Quantification of the mean fluorescence intensity (MFI) of SATB1 in PD-1^+^TIM-3^-^ Tpex versus PD-1^+^TIM-3^+^ Tex cells. *P < 0.05, ***P < 0.001, two-tailed unpaired *t*-test.

Through analyzing single-cell RNA sequencing (sc-RNA seq) data from human melanoma tissues in the GEO database (GSE115978), we revealed that *Satb1* was expressed in Tpex population, alongside well-established genes such as *Tcf7*, *Sell* and *Bach2* in this T cell subset, but was markedly downregulated in Tex-int and Tex-term cells (**Supplementary Figures 1A–D**). Furthermore, *Satb1* expression levels showed a positive correlation with *Tcf7*, *Sell* and *Bach2*, and a negative correlation with *Ifng* and *Pdcd1* in CD8^+^ TILs (**Supplementary Figure 1E**). To extend these findings, we established a B16 tumor-bearing mouse model and performed sc-RNA seq on isolated CD8^+^ TILs. Consistent with the results from human data, *Satb1* was expressed in Tpex cells but significantly downregulated in Tex cells (comprising Tex-int and Tex-term subsets) (**Figures 1A–D**). Additionally, *Satb1* expression correlated positively with *Tcf7* and *Sell*, and negatively with Pdcd1 and GzmB in mouse CD8^+^ TILs (**Supplementary Figure 1F**). To further assess SATB1 protein expression in tumor-infiltrating CD8^+^ T cells, we used a flow-cytometric gating strategy based on PD-1 and TIM-3 expression. Within CD8^+^ TILs, the PD-1^+^TIM-3^-^ population, which is enriched for progenitor-like exhausted cells and was operationally defined here as PD-1^+^TIM-3^-^ Tpex, exhibited significantly higher SATB1 expression than the PD-1^+^TIM-3^+^ population, which represents a more differentiated exhausted phenotype and was operationally defined here as PD-1^+^TIM-3^+^ Tex (**Figures 1E–G**). These observations align with the previously reported dynamics of *Satb1* expression in Tpex, Tex-int and Tex-term cells during chronic infection.

These data imply that the downregulation of *Satb1* appears to be closely associated with the differentiation of effector-like Tex-int cells.

### 2.2 SATB1 deficiency favored acquisition of a more differentiated exhausted phenotype under *in vitro* exhaustion-inducing conditions

**Figure 2:**
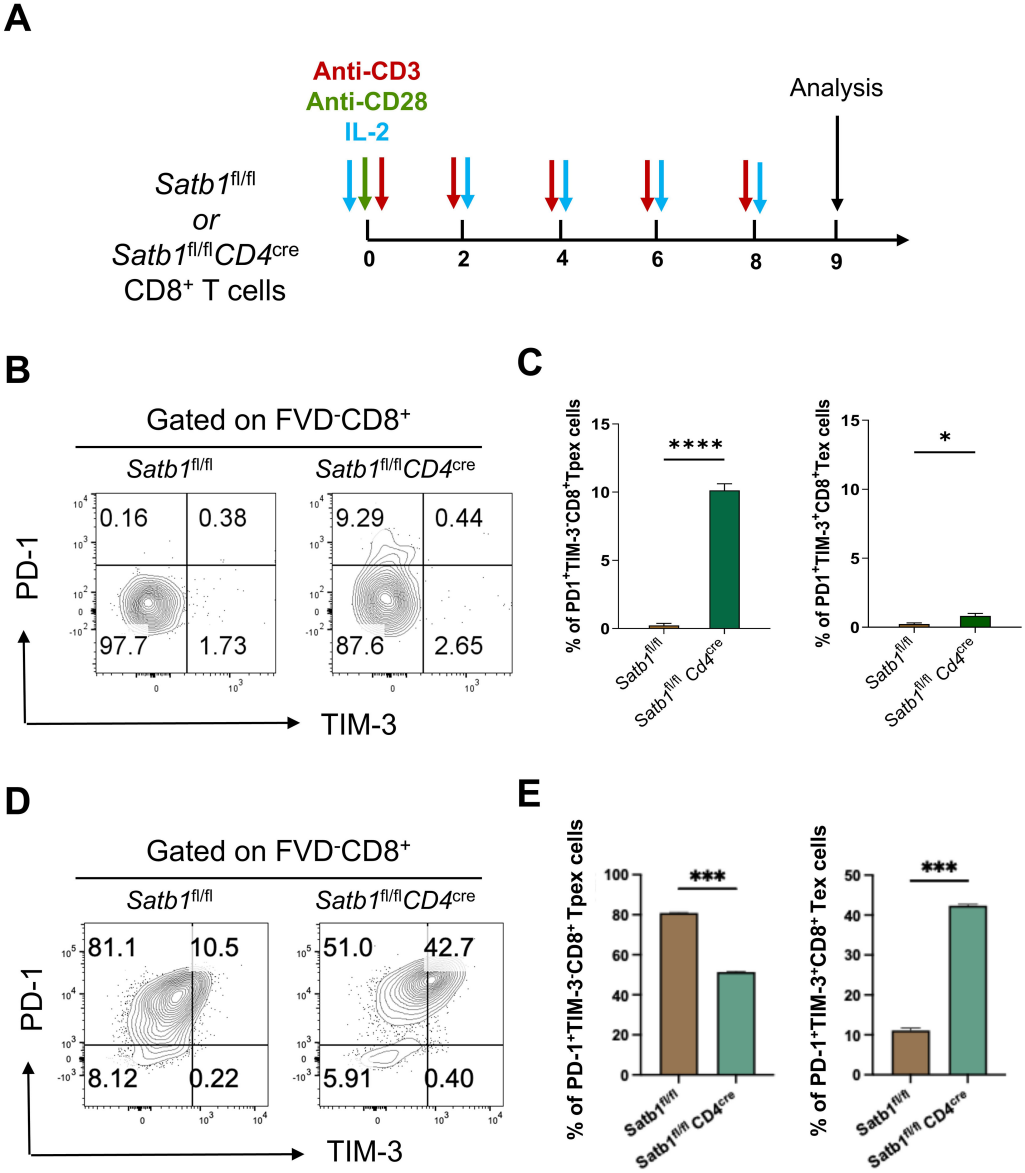
SATB1 deficiency favored acquisition of a more differentiated exhausted phenotype under *in vitro* exhaustion-inducing conditions. **(A)** Schematic diagram of the *in vitro* T cell exhaustion model *in vitro*. Naïve CD8^+^ T cells were isolated from the spleens of *Satb1*^fl/fl^*Cd4*^cre^ and *Satb1*^fl/fl^*Cd4*^wt^ mice and subjected to chronic antigen stimulation *in vitro*. **(B, C)** Baseline analysis of freshly isolated CD8^+^ T cells at day 0. **(B)** Representative flow-cytometry plots gated on FVD^-^CD8^+^ cells, showing the proportions of PD-1^+^TIM-3^-^ Tpex and PD-1^+^TIM-3^+^ Tex cells, as operationally defined in this analysis. **(C)** Quantification of the frequencies of PD-1^+^TIM-3^-^ and PD-1^+^TIM-3^+^ cells at day 0. **(D, E)** Analysis of CD8^+^ T cells after chronic antigen stimulation at day 9. **(D)** Representative flow-cytometry plots gated on FVD^-^CD8^+^ cells, showing the proportions of PD-1^+^TIM-3^-^ Tpex and PD-1^+^TIM-3^+^ Tex cells, as operationally defined in this analysis. **(E)** Quantification of the frequencies of PD-1^+^TIM-3^-^ and PD-1^+^TIM-3^+^ cells at day 9. FVD, fixable viability dye eFluor™ 780, was used to exclude dead cells. *P < 0.05, **P < 0.01, ***P < 0.001, ****P < 0.0001, ns, not significant; two-tailed unpaired *t*-test.

To investigate the role of SATB1 in Tex-int differentiation, we isolated naïve CD8^+^ T cells from the spleens of *Satb1*^fl/fl^*Cd4*^cre^ and *Satb1*^fl/fl^*Cd4*^wt^ mice and established an *in vitro* T cell exhaustion model following a previously reported protocol (**Figure 2A**). Compared to CD8^+^ T cells under acute antigen stimulation, those subjected to chronic antigen exposure in this model exhibited elevated expression of PD-1 and TIM-3 (**Supplementary Figure 2A**), confirming successful induction of T cell exhaustion. Notably, At baseline (day 0), both groups showed minimal PD-1^+^TIM-3^+^ cells, whereas Satb1-deficient CD8^+^ T cells exhibited a higher proportion of PD-1^+^TIM-3^-^ cells than control cells (**Figures 2B, C**). After chronic antigen stimulation, both groups acquired an exhausted phenotype; however, by day 9, Satb1-deficient cells displayed a lower proportion of PD-1^+^TIM-3^-^ cells together with a higher proportion of PD-1^+^TIM-3^+^ cells than control cells (**Figures 2D, E**). These findings indicate that, under *in vitro* exhaustion-inducing conditions, Satb1 deficiency favored a shift from a progenitor-like exhausted phenotype toward a more differentiated exhausted phenotype.

### 2.3 SATB1 deficiency restrained tumor progression and expanded CD8+ TILs

Given that the tumor microenvironment presents far greater complexity than *in vitro* T cell exhaustion models, we established B16 tumor-bearing models in *Satb1*^fl/fl^*Cd4*^cre^ and *Satb1*^fl/fl^*Cd4*^wt^ mice to define the impact of SATB1 deficiency in T cells on the maintenance and expansion of CD8^+^ T cells in antitumor immune responses.

**Figure 3:**
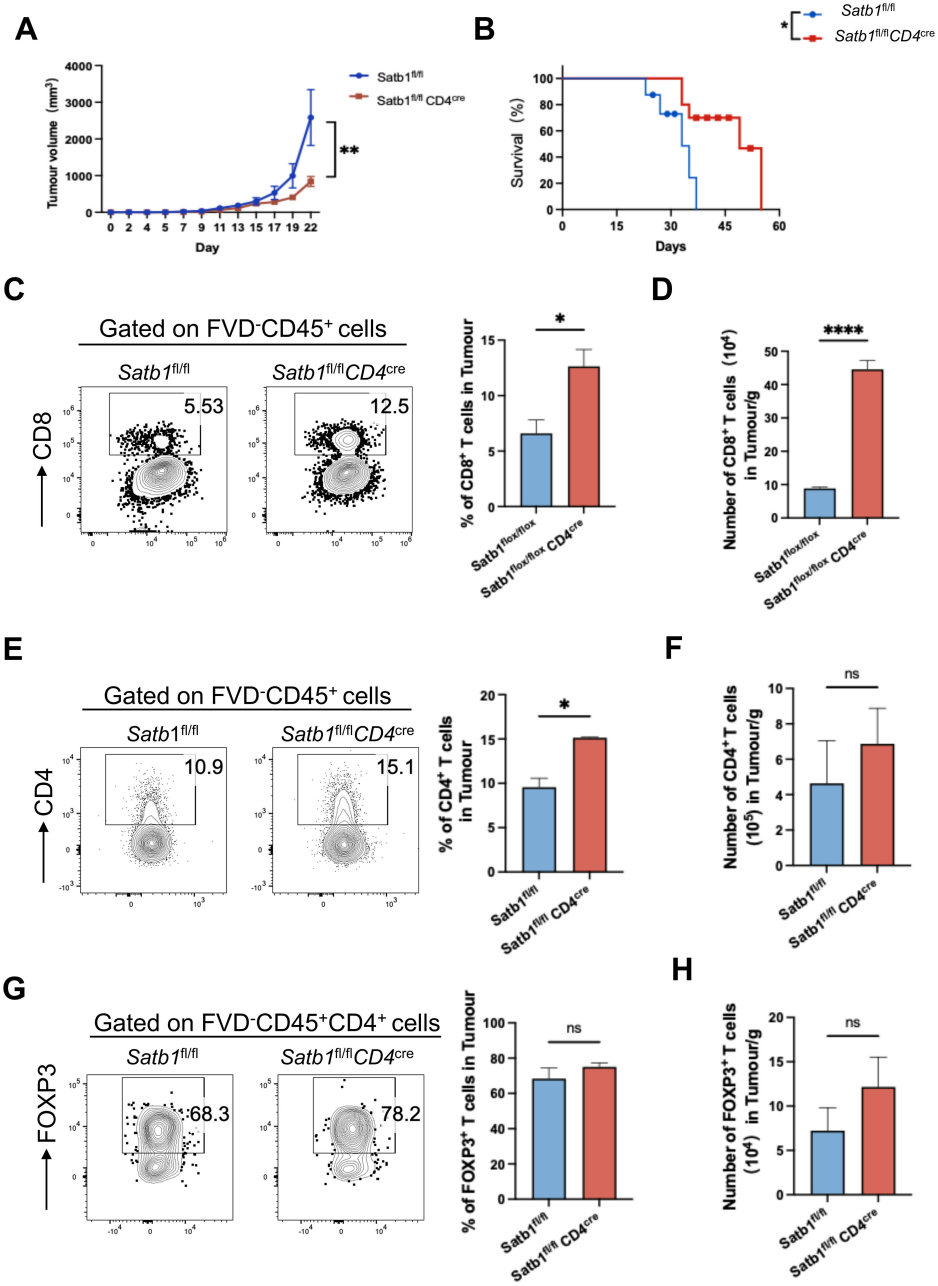
SATB1 deficiency restrained tumor progression and expands CD8^+^ TILs. *Satb1*^fl/fl^*Cd4*^cre^ and *Satb1*^fl/fl^*Cd4*^wt^ mice were subcutaneously inoculated with B16 melanoma cells. **(A)** Tumor growth curves measured over time (n = 6 mice per group). **(B)** Survival curves of the indicated tumor-bearing mouse cohorts. **(C, D)** Analysis of CD8^+^ T cell infiltration in tumors on day 12 post B16 cell inoculation. **(C)** Representative flow cytometry plots gated on live CD45^+^ cells showing CD8^+^ TILs (left) and quantification of the frequency (right). **(D)** Quantification of the absolute number of CD8^+^ TILs. **(E–H)** Analysis of CD4^+^ T cell and Foxp3^+^Tregs infiltration in tumors. **(E)** Representative flow cytometry plots gated on CD45^+^ cells showing CD4^+^ T cells (left) and quantification of the frequency (right). **(F)** Quantification of the absolute number of CD4^+^ T cells. **(G)** Representative flow cytometry plots gated on CD4^+^ cells showing Foxp3^+^Tregs (left) and quantification of the frequency (right). **(H)** Quantification of the absolute number of Foxp3^+^Tregs. *P < 0.05, **P < 0.01, ***P < 0.001, ****P < 0.0001, ns, not significant; two-tailed unpaired *t*-test or Log-rank test.

We first established that *Satb1* deficiency does not perturb T cell homeostasis, as the frequencies and numbers of splenic CD4^+^ T and CD8^+^ T cells in adult mice were unaltered (**Supplementary Figures 3A–D**). Strikingly, in tumor-bearing mice, *Satb1* deletion did not affect early tumor growth but strongly suppressed later-stage tumor progression, resulting in a significant survival advantage (**Figures 3A, B**).

Notably, although tumor size was unaffected at the early stage, *Satb1* deletion promoted robust immune infiltration, significantly increasing the frequency and number of both CD45^+^ cells and CD8^+^ T cells within tumors (**Supplementary Figures 3E, F**; **Figures 3C, D**). This enhanced T cell recruitment and/or expansion may be driven by the observed upregulation of T cell activation-associated transcription factor NFATc1 upon *Satb1* loss (**Supplementary Figure 4A**).

Furthermore, *Satb1* deficiency markedly upregulated PD-1 expression on CD8^+^ TILs (**Supplementary Figure 4B**), aligning with prior findings. This genetic alteration also led to an elevated abundance of PD-1^+^ T cells within tumors, while reducing their presence in draining lymph nodes (**Supplementary Figures 4C, D**). Moreover, although *Satb1* loss increased both the frequency of CD4^+^ T cells infiltrating tumors, it did not significantly affect the proportion and quantity of Foxp3^+^ regulatory T cells (Tregs) among this population (**Figures 3E–H**).

### 2.4 SATB1 deficiency promoted the generation of effector-like CD8^+^ Tex-int cells during early-stage of tumor progression

While our *in vitro* data suggested that *Satb1* deletion enhances Tpex-to-Tex differentiation, precisely how this process is regulated by SATB1 remained unclear and required *in vivo* investigation.

**Figure 4:**
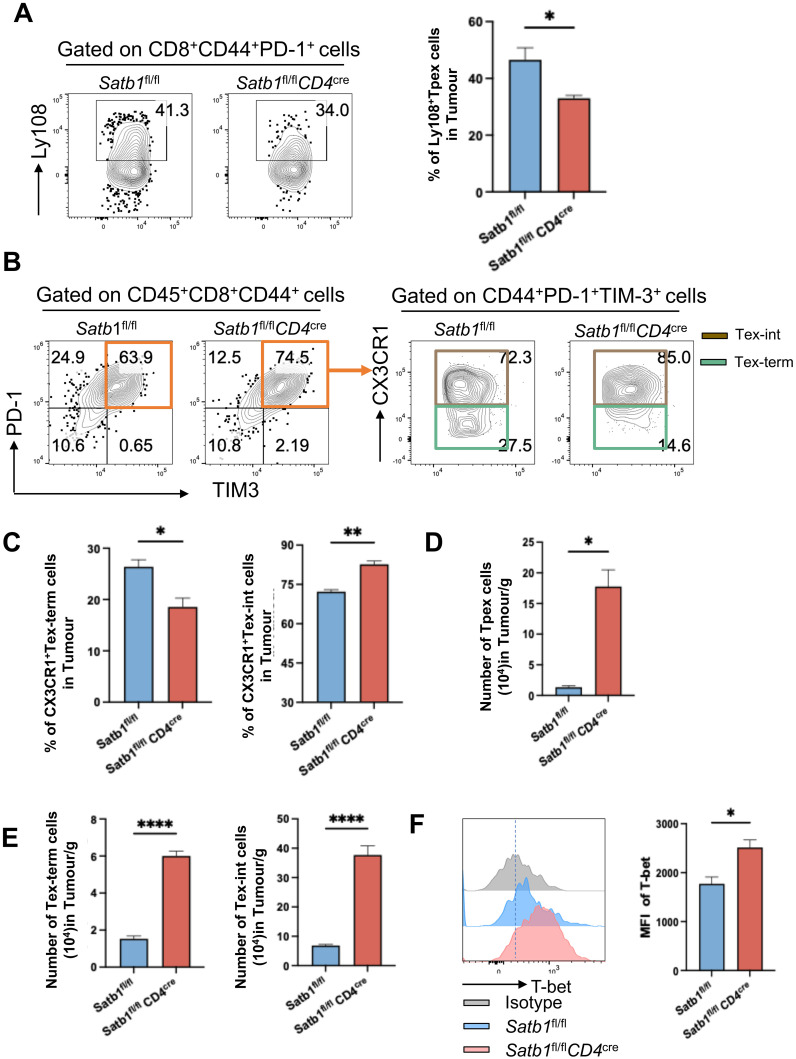
SATB1 deficiency promoted the generation of effector-like CD8^+^ Tex-int cells during the early stage of tumor progression. Analysis of intratumoral CD8^+^ T cell subsets in *Satb1*^fl/fl^*Cd4*^cre^ and *Satb1*^fl/fl^*Cd4*^wt^ mice on day 12 post tumor cell inoculation. **(A)** Gating strategy for identifying Ly108^+^Tpex subset from CD8^+^ TILs (left) and quantification of the frequency (right). **(B)** Gating strategy for identifying CX3CR1^+^Tex-int and CX3CR1^-^Tex-term subsets from CD8^+^ TILs. **(C)** Quantification of the frequency CX3CR1^+^Tex-int and CX3CR1^-^Tex-term subsets. **(D)** Quantification of the absolute number of Tpex cells. **(E)** 2Quantification of the absolute number of CX3CR1^+^Tex-int and CX3CR1^-^Tex-term subsets. **(F)** Representative flow plot of T-bet MFI and the quantification. *P < 0.05, **P < 0.01, ***P < 0.001, ****P < 0.0001, ns, not significant; two-tailed unpaired *t*-test.

Analysis of intratumoral CD8^+^ T cells during early-stage of tumor progression (day 12) revealed that *Satb1* deficiency altered Tex subset distribution. It reduced the proportions of Ly108^+^Tpex and Ly108^-^CX3CR1^-^Tex-term populations while significantly increasing the frequency of CX3CR1^+^ Tex-int cells (**Figures 4A–C**). Concurrently, the numbers of all three subsets were elevated (**Figures 4D, E**). Furthermore, this shift toward an effector phenotype was accompanied by significant upregulation of the key transcription factor T-bet (**Figure 4F**).

We next assessed key effector-associated molecules in CD8^+^ TILs, including CD107α, IFN-γ, and granzyme B (GzmB) (**Supplementary Figures 4E–G**). Notably, although Satb1 deficiency increased both the proportion and absolute number of CX3CR1^+^ Tex-int cells, these cells exhibited reduced expression of CD107α, IFN-γ, and GzmB. Together with the elevated PD-1 expression observed after Satb1 loss (**Supplementary Figure 4D**), these findings suggest that Satb1 deficiency promotes the accumulation of Tex-int-like cells while impairing the acquisition or maintenance of their full effector program. Thus, phenotypic expansion of this population is not accompanied by enhancement of cytotoxic function.

### 2.5 The effector-like CD8^+^ Tex-int cells were sustained in the tumor microenvironment of SATB1 deficient-mice during late-stage of tumor progression

While *Satb1* deficiency increased the abundance of CD8^+^ TILs during early tumorigenesis, it compromised their effector capacity, potentially explaining the absence of significant differences in tumor progression between *Satb1*^fl/fl^*Cd4*^cre^ and *Satb1*^fl/fl^*Cd4*^wt^ mice at this phase. In contrast, the tumor progression was markedly slower in *Satb1*^fl/fl^*Cd4*^cre^ mice during late-stage of tumor progression. To investigate the basis for this disparity, we performed a detailed analysis of the compositional and functional landscape of exhausted CD8^+^ T cell subsets in advanced tumors.

**Figure 5:**
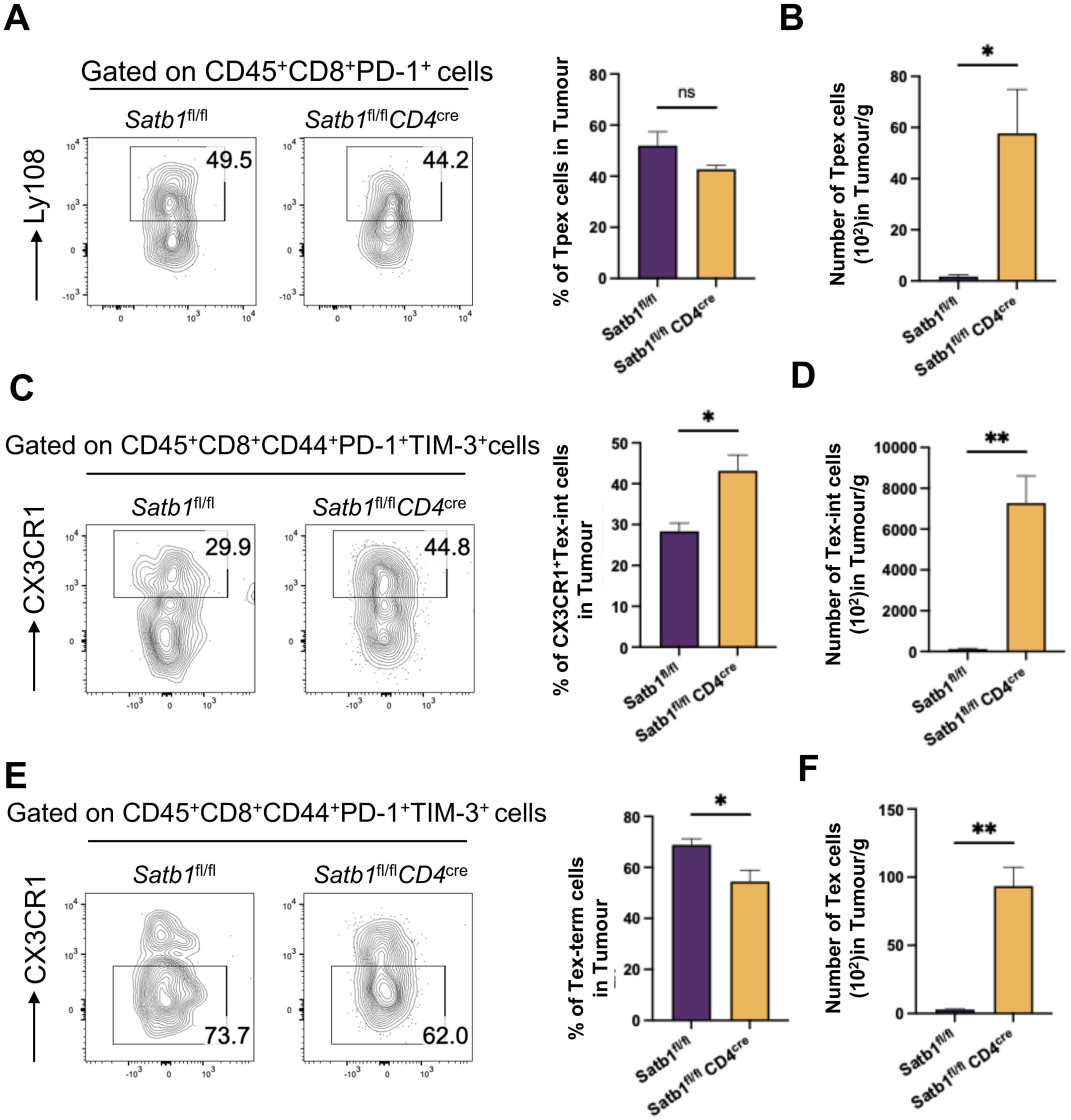
CD8^+^ Tex-int cells were sustained in the tumor microenvironment of SATB1-deficient mice during the late stage of tumor progression. Analysis of intratumoral CD8^+^ T cell subsets in *Satb1*^fl/fl^*Cd4*^cre^ and *Satb1*^fl/fl^*Cd4*^wt^ mice on day 12 post tumor cell inoculation. **(A, B)** Analysis of Tpex cells: **(A)** frequency and **(B)** absolute number. **(C, D)** Analysis of Tex-int cells: **(C)** frequency and **(D)** absolute number. **(E, F)** Analysis of Tex-term cells: **(E)** frequency and **(F)** absolute number. *P < 0.05, **P < 0.01, ns, not significant; two-tailed unpaired *t*-test.

Evaluation of late-stage tumors (day 22) revealed that *Satb1*^fl/fl^*Cd4*^cre^ mice maintained significantly higher frequency and number of CD8^+^ TILs compared to *Satb1*^fl/fl^*Cd4*^wt^ controls (**Supplementary Figures 5A, B**). Interestingly, while *Satb1* deficiency did not alter the proportion of Ly108^+^Tpex cells, it strikingly increased their absolute abundance at this stage (**Figures 5A, B**). Importantly, *Satb1* loss led to a concurrent rise in both the frequency and number of CX3CR1^+^Tex-int cells (**Figures 5C, D**). Furthermore, although the frequency of terminally exhausted Tex-term cells was reduced in *Satb1*^fl/fl^*Cd4*^cre^ mice (**Figure 5E**), their number remained elevated (**Figure 5F**). Of note, CD8^+^ TIL effector function at this late stage was comparable between *Satb1*^fl/fl^*Cd4*^cre^ and *Satb1*^fl/fl^*Cd4*^wt^ controls (**Supplementary Figures 5C, D**).

Taken together, these late-stage findings demonstrate that *Satb1* deficiency sustains CD8^+^ TILs and promotes the accumulation of effector-like Tex-int subsets while preserving their effector-associated molecule expression at a level comparable to the control.

### 2.6 SATB1 deficiency promoted the differentiation of T_TSM_ cells toward Tpex cells in tumor-draining lymph nodes

T_TSM_ cells function as self-renewing upstream precursors to Tpex cells (10, 25, 26). Upon chronic antigen exposure, T_TSM_ cells commit to CD62L^+^ or CD62L^-^ Tpex lineages and initiate migration into tumors, where they differentiate into effector Tex-int cells to eliminate malignancies (18, 27, 28). Therefrom, we sought to examine the influence of *Satb1* deficiency on T_TSM_ and Tpex subsets in TdLNs.

**Figure 6:**
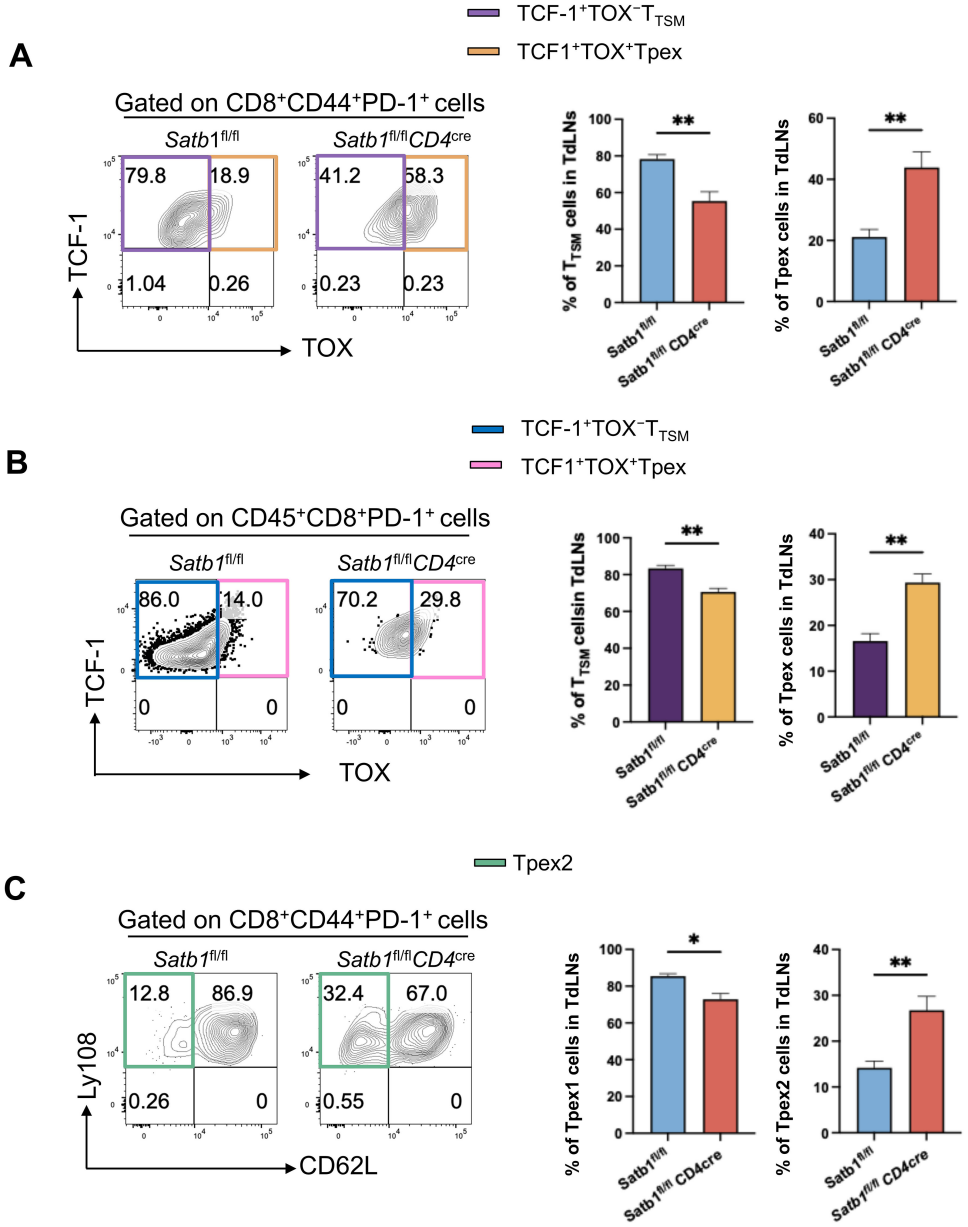
SATB1 deficiency promoted the differentiation of T_TSM_ cells toward Tpex cells in TdLNs. **(A, B)** Flow cytometric analysis of T cell subsets in TdLNs on day 12 **(A)** and day 22 **(B)** post tumor cell inoculation. **(A, B)** left, representative flow cytometry plots showing TCF-1 and TOX expression to identify TCF-1^+^TOX^-^T_TSM_ and TCF-1^+^TOX^+^Tpex cells. **(A, B)** Quantification of the frequencies of T_TSM_ (left) and Tpex (right) cells. **(C)** Analysis of Tpex subsets in TdLNs on day 12 post tumor cell inoculation. Left: Representative flow cytometry plots of CD62L expression on TCF-1^+^TOX^+^ Tpex cells, defining CD62L^+^ Tpex1 and CD62L^-^ Tpex2 subsets. Right: Quantification of the frequencies of Tpex 1 an Tpex2 cells among total Tpex cells. *P < 0.05, **P < 0.01; two-tailed unpaired *t*-test.

We first examined T cells in the inguinal lymph nodes of tumor-free mice and found that *Satb1* deficiency did not significantly affect the proportion and abundance of CD45^+^ population and T cells (**Supplementary Figures S6A-C**). Following previous reports, T cell subsets in TdLN of tumor-bearing mice were identified based on TCF-1 and TOX expression, with TCF-1^+^TOX^-^ cells classified as TTSM cells and TCF-1^+^TOX^+^ cells as Tpex cells. The Tpex population was further subdivided according to CD62L expression into CD62L^+^Tpex (Tpex1) and CD62L^-^Tpex (Tpex2) subsets. Using these established phenotypic criteria, we found that Satb1 deletion significantly reduced the frequency of T_TSM_ cells while concomitantly increasing the frequency of Tpex cells in TdLNs at both the early (day 12) and late (day 22) stages of tumor progression, compared with controls (**Figures 6A, B**). Tpex2 cells are recognized for their strong proliferative and migratory capabilities and can further differentiate into effector-like Tex-int cells that migrate from TdLN to tumor tissue to mediate cytotoxic functions. Notably, at the early tumor stage (day 12), *Satb1* deficiency significantly promoted the differentiation of Tpex1 into Tpex2 cells (**Figure 6C**).

Collectively, Satb1 deficiency reshapes T cell landscape in TdLNs by reducing T_TSM_ frequency, expanding Tpex pool and preferentially driving Tpex1-to-Tpex2 differentiation at the early tumor stage.

## 3 Discussion

Under persistent antigen exposure in tumors, CD8^+^ T cells adopt an exhausted state, a distinct transcriptional and epigenetic program characterized by impaired effector function, dysregulated homeostasis and upregulated inhibitory receptors (37). Despite this attenuation, they retain partial anti-tumor activity. The exhausted T cell pool is maintained by TCF-1^+^Tpex cells, a self-renewing stem-like subset (21, 33). This population can be subdivided into CD62L^+^Tpex1 cells with strong self-renewal capacity and CD62L^-^Tpex2 cells, which demonstrate greater proliferative ability, enter circulation, and further differentiate into effector-like Tex-int cells that migrate to tumor sites (38).

The hierarchical differentiation of exhausted CD8^+^ T cells (T_TSM_→Tpex1→Tpex2→Tex-int→Tex-term) culminates in the Tex-int subset, which serves as the key antitumor effector by secreting IFN-γ/GzmB/TNF-α to kill targets (14, 39, 40). Enhancing Tex-int function is a central goal of checkpoint blockade, prompting this study to investigate how *Satb1* regulates its differentiation.

SATB1, a global chromatin organizer, was demonstrated to regulate stem-like CD8^+^ T cells (35, 41, 42). Here, we observed lower SATB1 expression in Tex-int compared to Tpex cells in tumor-bearing mice, suggesting the potential role of SATB1 timely downregulation in Tex-int cell differentiation from Tpex subset. This was confirmed under exhaustion-inducing conditions *in vitro* in the current study, where *Satb1* deficiency reduced Tpex proportion while increasing Tex cells, indicating accelerated differentiation.

Further, we found that *Satb1* deletion significantly expanded CD8^+^ TILs without affecting early tumor growth. Detailed analysis revealed enhanced Tpex-to-Tex-int differentiation in tumors, increasing Tex-int proportion and number. In TdLNs, *Satb1* deficiency shifted differentiation dynamics: T_TSM_ decreased while Tpex increased, particularly the CD62L^-^ Tpex2 subset that is a population with known migratory potential that differentiates into tumoricidal Tex-int cells (10, 43). These findings support the notion that Satb1 loss facilitates progression along the exhausted CD8^+^ T-cell differentiation hierarchy in both tumors and TdLNs. At the same time, the reduction in the TTSM compartment raises the possibility that accelerated differentiation may come at the cost of depleting an upstream reservoir with stem-like properties. Such a trade-off may not be detrimental in the setting of established tumor control examined here, but it could potentially limit long-term persistence, recall capacity, or protection against tumor recurrence, which will require further investigation in future studies.

Extending observation to late tumor stage, we found that *Satb1* deficiency ultimately suppressed tumor growth, correlating with persistently increased CD8^+^ T cell infiltration and Tex-int differentiation in tumors. In early tumors, although the CX3CR1^+^ Tex-int pool was expanded, these cells displayed reduced expression of effector-associated molecules, indicating that phenotypic accumulation was not initially matched by full functional competence. By the late stage, the overall antitumor effect observed in Satb1-deficient mice may therefore reflect sustained accumulation and increased total abundance of Tex-int cells, rather than unequivocal restoration of per-cell effector function. Collectively, our findings demonstrate that *Satb1* deletion reshapes T cell differentiation, promotes the generation and sustained accumulation of effector-like subsets, and strengthens antitumor immunity, providing a rationale for novel immunotherapeutic strategies.

Notwithstanding these findings, our study has limitations. First, the precise mechanisms by which SATB1 regulates key transcription factors in distinct T-cell subsets during tumor immunity remain to be elucidated. Second, the constitutive Satb1 deletion in CD4^+^ T cells in our model may complicate the interpretation of the observed phenotype, as SATB1 could intrinsically shape CD4^+^ T-cell differentiation and function, a possibility only preliminarily addressed here. Accordingly, although our data support a role for Satb1 in remodeling exhausted CD8^+^ T-cell differentiation, they do not formally establish that all of the observed effects are CD8-intrinsic. Future studies using adoptive transfer approaches or more selective CD8-targeted models will be required to isolate the cell-intrinsic contribution of Satb1 loss in CD8^+^ T cells. Third, despite expanding the CX3CR1^+^Tex-int pool, Satb1 ablation impaired its effector function at the early stage of tumor progression, pointing to an unresolved regulatory role that might be addressed by future combination therapies with cytokines.

In summary, our study delineates an unappreciated role for SATB1 in regulating the differentiation dynamics of exhausted CD8^+^ T cells during antitumor responses. We demonstrate that SATB1 expression is dynamically regulated during T cell exhaustion, with its downregulation facilitating the generation of effector-like Tex-int cells. Genetic ablation of *Satb1* promotes the differentiation and accumulation of Tex-int cells while reshaping the T cell landscape in both tumor microenvironment and draining lymph nodes. Although early functional impairment was observed, *Satb1* deficiency ultimately enhanced antitumor immunity through sustained Tex-int maintenance. These findings position SATB1 as a molecular gatekeeper of T Cell differentiation and suggest that therapeutic modulation of SATB1 activity, particularly in combination with strategies to maintain T cell function, may provide a novel approach to improve cancer immunotherapy outcomes.

## 4 Materials and methods

### 4.1 Animals and tumor model

All animal experiments were performed using 6–8-week-old C57BL/6j female mice with strict age matching within experimental groups. Procedures were conducted in a specific pathogen-free (SPF) facility approved by the Soochow University Animal Ethics Committee (Approval ID: 202412A0609). *Satb*1^fl/fl^*Cd4*^cre^ mice and their littermate controls *Satb*1^fl/fl^*Cd4*^wt^ (*Satb*1^fl/fl^) were bred. The conditional knockout mice were established on a C57BL/6j background, purchased from GemPharmatech Co., Ltd. (Jiangsu, China). Mice were housed under standardized conditions with a 12:12-hour light-dark cycle in open-top cages, provided ad libitum access to food and water. Health monitoring was performed routinely, with intensified surveillance when anticipating adverse effects. B16 melanoma cells at a density of 1.0 × 10^5^ cells/50 µL were subcutaneously inoculated into C57BL/6J mice. Tumor size was monitored at 2 to 3-day intervals, and tumor growth curves were plotted.

### 4.2 Sample collection and processing

Tumor-bearing mice were euthanized on day 12 or 22 post inoculation of B16 cells. Single-cell suspensions were prepared from tumors, tumor-draining lymph nodes (TdLNs) and spleens to analyze the phenotype and activity of exhausted CD8^+^ T-cell subsets. For tumor tissues, samples were placed in 2 mL of cold RPMI 1640 medium without FBS, mechanically dissociated with surgical scissors, and minced into small fragments. The tissue fragments were digested in serum-free RPMI 1640 containing DNase I (0.33 mg/mL; Sigma-Aldrich) and Liberase TL (0.25 mg/mL; Roche) at 37.5 °C for 30 minutes. The digested tissue was transferred to a 70-µm cell strainer and gently pressed through using the flat end of a 1-mL syringe plunger. Lymphocytes were then isolated by Percoll (Cytiva) density-gradient centrifugation. TdLNs were harvested and gently ground between the frosted edges of two sterile glass slides. The resulting suspension was filtered through a 200-mesh sieve into a 96-well V-bottom plate and kept on ice. For spleens, tissues were mechanically dissociated and passed through a cell strainer, and red blood cells were lysed using ACK Lysing Buffer (Gibco). The cell suspension was subsequently kept on ice for further use.

### 4.3 scRNA-seq

The tumor cell suspension was incubated on ice for 20 minutes with a biotinylated anti-CD8 antibody, followed by a 15-minute incubation with anti-biotin MicroBeads (Miltenyi Biotec). CD8^+^ T cells were then isolated by magnetic separation using LS MACS columns (Miltenyi Biotec), after which libraries were prepared and sequencing was performed. Raw sequencing data were returned for analysis in R, including quality control, normalization, identification of the top 2,000 highly variable genes, and scaling. To ensure a highly pure CD8^+^ compartment, cells were further enriched computationally using scGateModels. Principal component analysis (PCA; npcs = 50) was computed, and the first 20 principal components (dims = 1:20) were used for downstream dimensionality reduction and graph-based clustering (resolution = 0.7). Differential expression was assessed for each subcluster versus all remaining subclusters using FindMarkers, and four biologically meaningful subpopulations were annotated based on canonical marker genes.

Human scRNA-seq data from GEO (GSE115978) were analyzed using R (4.4.2) and Seurat (v5). CD8^+^ T cells were first subsetted using the provided cell-type annotations, followed by normalization and scaling with the standard Seurat workflow, including identification of highly variable features. Dimensionality reduction was performed using PCA followed by UMAP to generate the embeddings shown.

### 4.4 Flow cytometry

Immune cell subsets in tumor samples were identified by flow cytometry, and data were acquired on a flow cytometer (Beckman, CA, USA). Surface staining was performed with the specified antibodies for 20 minutes according to the manufacturer’s instructions. For intracellular detection of IFN-γ and TNF-α in CD8^+^ tumor-infiltrating lymphocytes (TILs), harvested cells were stimulated with PMA (10 ng/mL) and ionomycin (1 µg/mL) for 1 hour, followed by incubation with brefeldin A (10 µg/mL) for an additional 3 hours. Intracellular cytokine staining was performed using antibodies specific for IFN-γ and TNF-α. All fluorochrome-conjugated antibodies were sourced from BioLegend as follows: FITC-conjugated anti-mouse CD45(clone 30-F11), PerCP/Cy5.5-conjugated anti-mouse CD8α(clone 53-6.7), PerCP/Cy7–conjugated anti-CD4(clone GK1.5), PE-conjugated anti-mouse PD-1(clone 29F.1A12), APC-conjugated anti-mouse Ly108 (clone 30-AJ), BV785-conjugated anti-mouse CD62L (clone MEL-14), BV421-conjugated anti-mouse CX3CR1 (clone SA011F11), BV605-conjugated anti-mouse TIM-3 (clone RMT3-23),PE-conjugated anti-mouse TOX (clone TXRX10),FITC-conjugated anti-mouse IFN-γ (clone XMG1.2), PE-conjugated anti-mouse GZMB (clone NGZB), PerCP/Cy5.5-conjugated anti-mouse TNF-α (MP6-XT22), Pacific Blue-conjugated anti-mouse Foxp3 (clone MF-14) and AF488-conjugated anti-mouse SATB1 (clone O96C6). Fixable Viability Dye eFluor™ (FVD) 780 was obtained from Thermo Fisher Scientific. Flow cytometry data were acquired on the CytoFLEX platform and analyzed using FlowJo V10.0 software (Tree Star Inc., CA, USA).

### 4.5 Statistical analysis

Flow cytometry data were analyzed using FlowJo v10.0 (Tree Star, Ashland, OR, USA). Statistical analyses for experimental data were performed using GraphPad Prism 8.0 (GraphPad Software, San Diego, CA, USA). Single-cell RNA-seq data were analyzed in R (version 4.4.2) using the Seurat package. Data are presented as mean ± SEM unless otherwise indicated. Comparisons between two groups were performed using a two-tailed unpaired Student’s t test. Tumor growth curves were analyzed by two-way ANOVA, and survival curves were analyzed using the Kaplan–Meier method with the log-rank test. A *P* value < 0.05 was considered statistically significant. Error bars in all figures represent mean ± SEM.

## Data Availability

The raw data supporting the conclusions of this article will be made available by the authors, without undue reservation.

## Supplementary material

Figure S1 Analysis of Satb1 expression in human and mouse exhausted CD8^+^ T subsets. **(A-D)** Analysis of human scRNA-seq data from melanoma tissues (GEO: GSE115978). **(A)** UMAP projection of CD8⁺ TIL subsets. (B) Dot plot showing the expression of key genes across human CD8^+^ TIL subsets. **(C)** UMAP feature plots showing the expression of Satb1. **(D)** Violin plot showing the expression levels of Satb1 across CD8^+^ T cell subsets **(E)** Correlation analysis of Satb1 expression with the indicated genes in human CD8^+^ TILs. **(F)** Correlation analysis of Satb1 expression with the indicated genes in mouse CD8^⁺^ TILs from B16 tumors.

Figure S2 Validation of the in vitro T cell exhaustion model. Flow cytometric analysis confirming elevated PD-1 and TIM-3 expression on CD8^+^ T cells under chronic antigen stimulation (exhaustion model) compared to acute stimulation.

Figure S3 SATB1 deficiency did not alter steady-state T cell homeostasis while it enhanced tumor immune infiltration. **(A-D)** Analysis of splenic T cells in tumor-free adult Satb1fl/flCd4cre and Satb1fl/flCd4wt mice. Representative flow cytometry plots **(A)** and quantification of the frequency of CD4^+^ and CD8^+^ T cells among CD45+ cells **(B)**. **(C, D)** Quantification of the absolute numbers of **(C)** CD4^+^ T and **(D)** CD8^+^ T cells in the spleen. **(E, F)** Assessment of total immune cell infiltration in tumors on day 12 post-inoculation of tumor cells. Quantification of the frequency **(E)** and absolute number of CD45⁺ cells **(F)**. *P < 0.05, **P < 0.01, ns, not significant; two-tailed unpaired t-test.

Figure S4 Impact of SATB1 deficiency on T cell activation, PD-1 expression and early effector function. **(A)** Determination and quantification of NFATc1 expression in CD8⁺ TILs from tumors on day 12. **(B)** Determination and quantification of PD-1 expression on total CD8⁺ TILs. **(C, D)** Analysis of PD-1^+^ T cell frequency among CD8⁺ T cells in tumors **(C)** and TdLNs **(D)**. **(E-G)** Functional analysis of CD8^⁺^ TILs from tumors on day 12. Determination and quantification of GzmB **(E)**, CD107a **(F)** and IFN-γ **(G)** levels in CD8⁺ TILs. *P < 0.05, ** *P < 0.001; two-tailed unpaired t-test.

Figure S5 Sustained T cell infiltration and function in late-stage tumors of SATB1-deficient mice. Analysis of tumors from Satb1fl/flCd4cre and Satb1fl/flCd4wt mice on day 22 post-inoculation of tumor cells. **(A)** Determination and quantification the frequency of CD8^+^ TILs. **(B)** Quantification of the absolute number of CD8⁺ TILs. **(C, D)** Functional analysis of CD8^+^ TILs at the late stage. Determination and quantification of IFN-γ **(C)** and GzmB **(D)** levels in CD8^+^ TILs. **P < 0.01, ***P < 0.001, ns, not significant; two-tailed unpaired t-test.

Figure S6 SATB1 deficiency did not affect steady-state T cell populations in lymph nodes. Analysis of inguinal lymph nodes from tumor-free Satb1fl/flCd4cre and Satb1fl/flCd4wt mice. **(A)** Representative flow cytometry plots and quantification of the frequency and absolute number of CD45^+^ cells. **(B)** Quantification of the frequency and absolute number of CD8^+^ T cells. **(C)** Quantification of the frequency and absolute number of CD4^+^ T cells. ns, not significant; two-tailed unpaired t-test.
