# Supplementary material for: Ablating Satb1 reprograms the differentiation trajectory of exhausted CD8+ T subsets to enhance antitumor immunity
Source: Front Immunol. 2026 Jun 22;17:1744549. doi: 10.3389/fimmu.2026.1744549 (PMC13333729; doi:10.3389/fimmu.2026.1744549)
Supplement: Figure S1 — Analysis of Satb1 expression in human and mouse exhausted CD8+ T subsets. (A-D) Analysis of human scRNA-seq data from melanoma tissues (GEO: GSE115978). (A) UMAP projection of CD8⁺ TIL subsets. (B) Dot plot showing the expression of key genes across human CD8+ TIL subsets. (C) UMAP feature plots showing the expression of Satb1. (D) Violin plot showing the expression levels of Satb1 across CD8+ T cell subsets (E) Correlation analysis of Satb1 expression with the indicated genes in human CD8+ TILs. (F) Correlation analysis of Satb1 expression with the indicated genes in mouse CD8⁺ TILs from B16 tumors. [file Presentation1.pptx]

## Slide 1
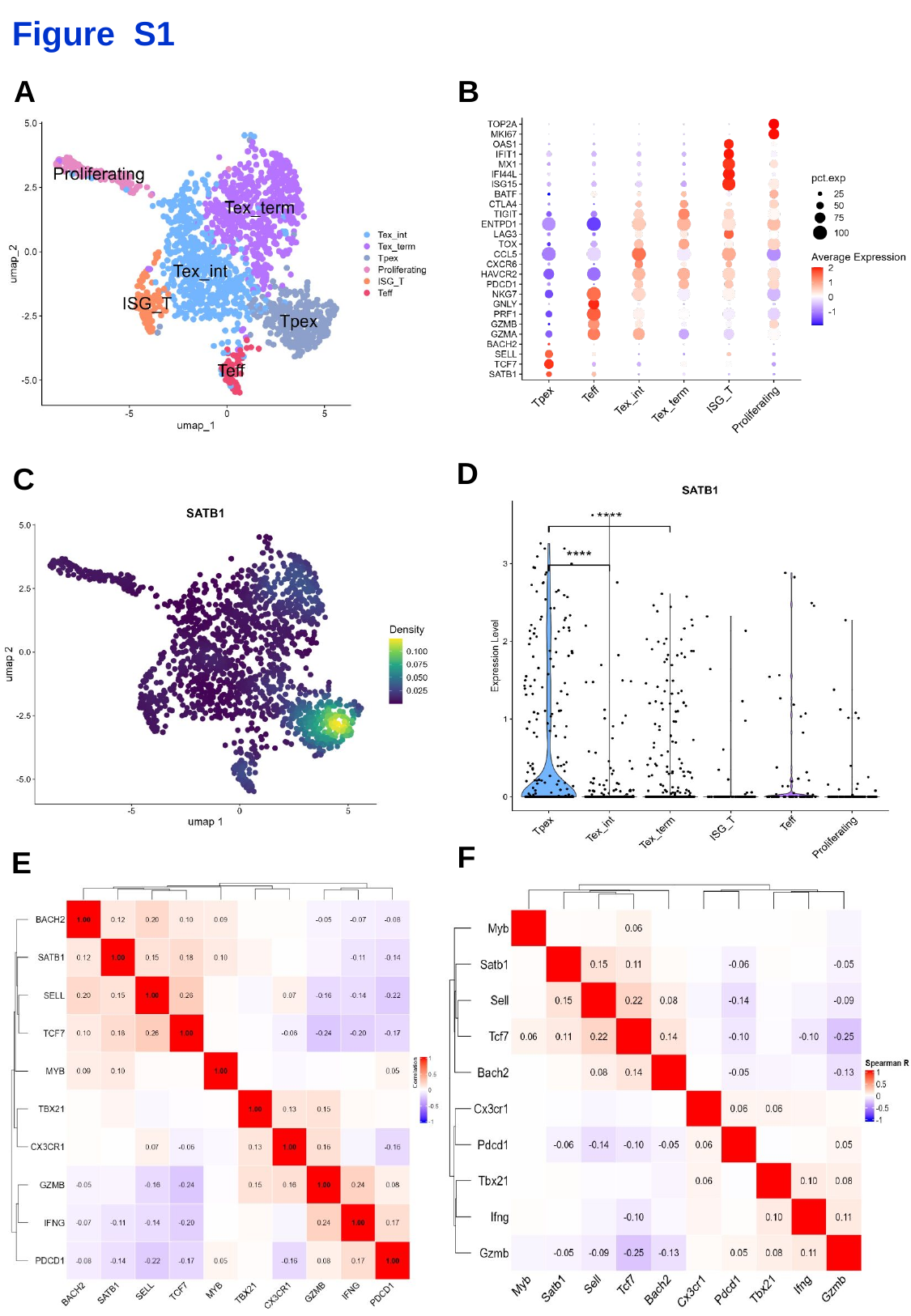

Figure S1
A
B
D
C
F
E

## Slide 2
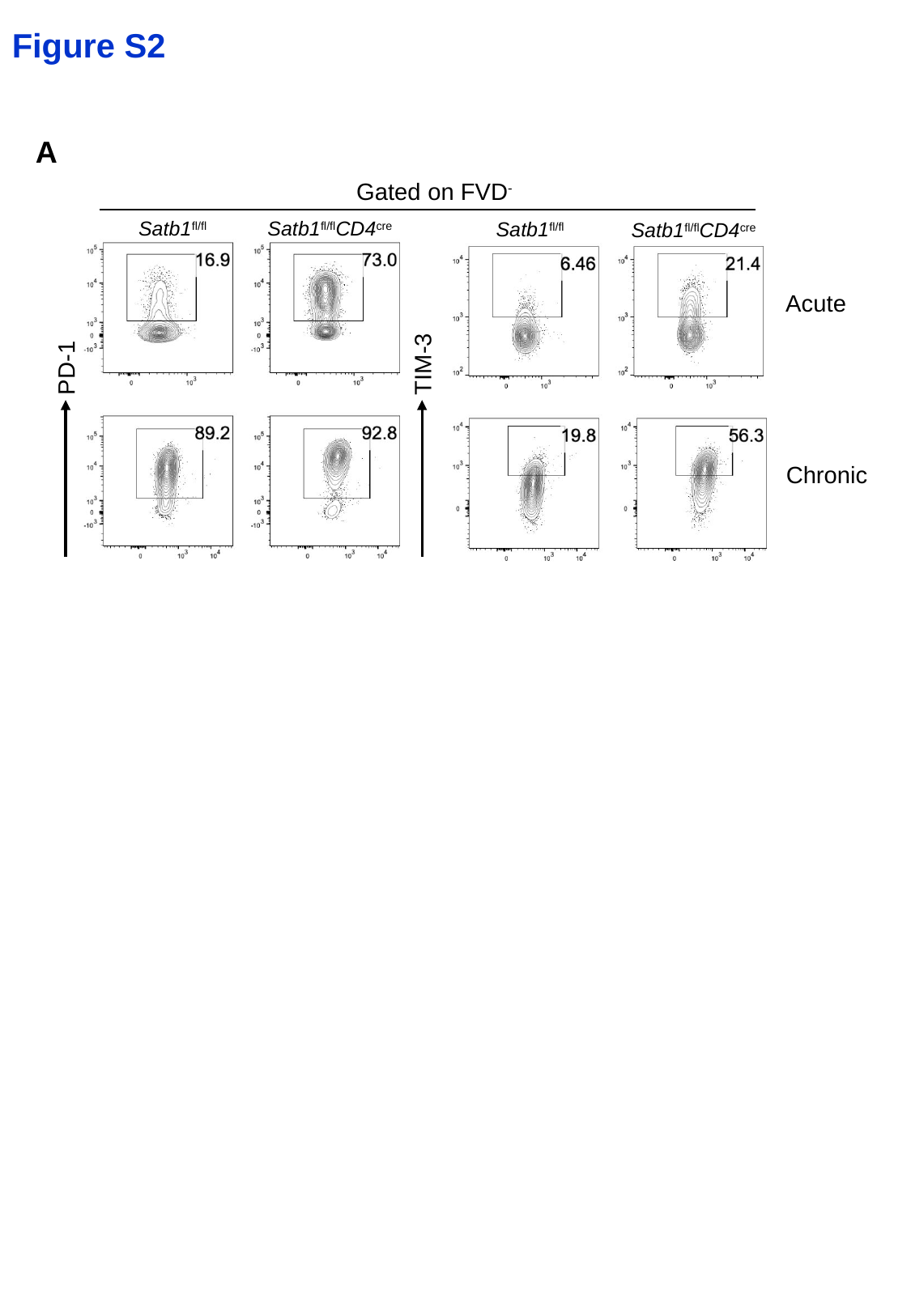

Figure S2
A
Gated on FVD-
Satb1fl/fl
Satb1fl/flCD4cre
Satb1fl/fl
Satb1fl/flCD4cre
Acute
PD-1
TIM-3
Chronic

## Slide 3
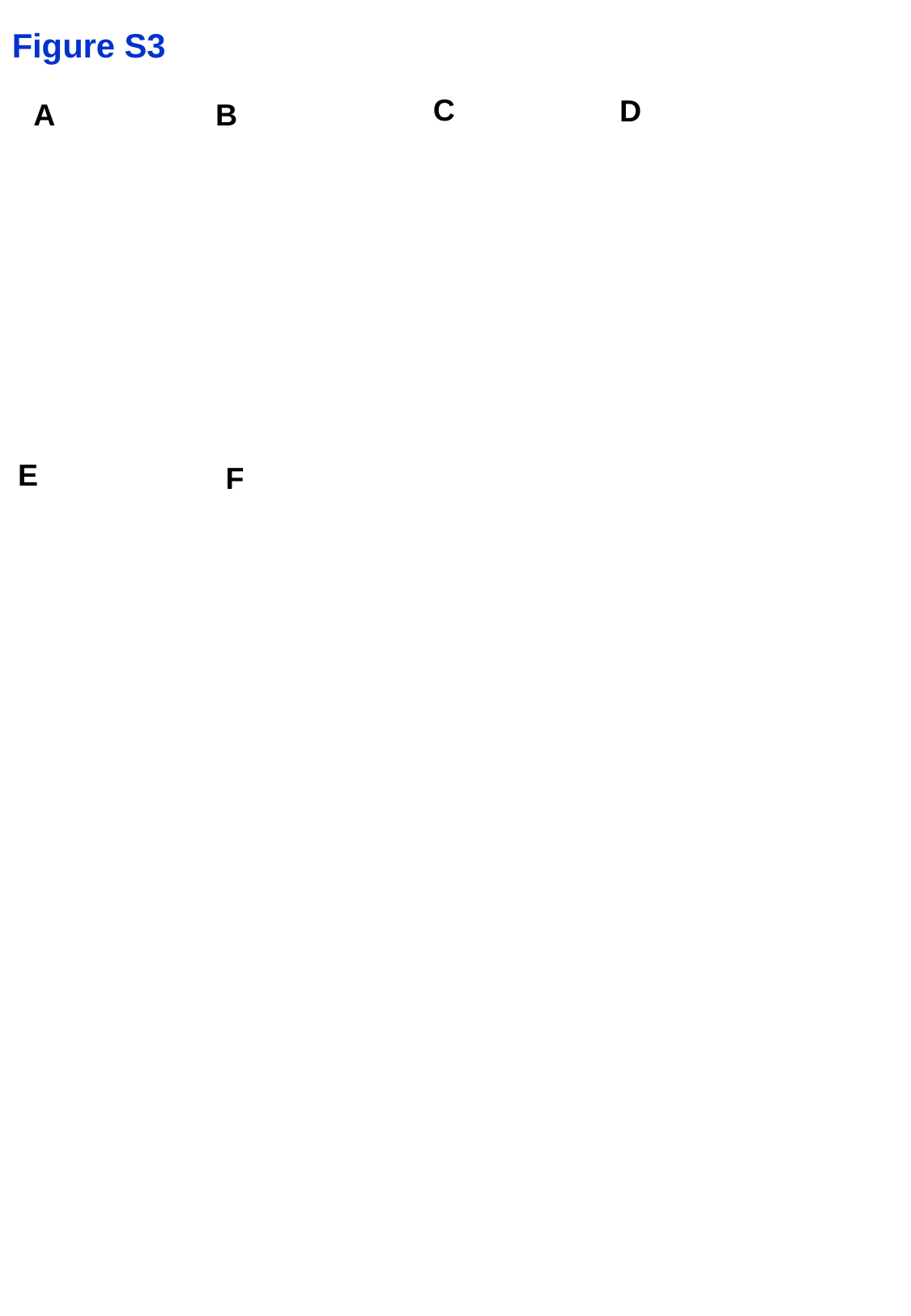

Figure S3
C
D
A
B
E
F

## Slide 4
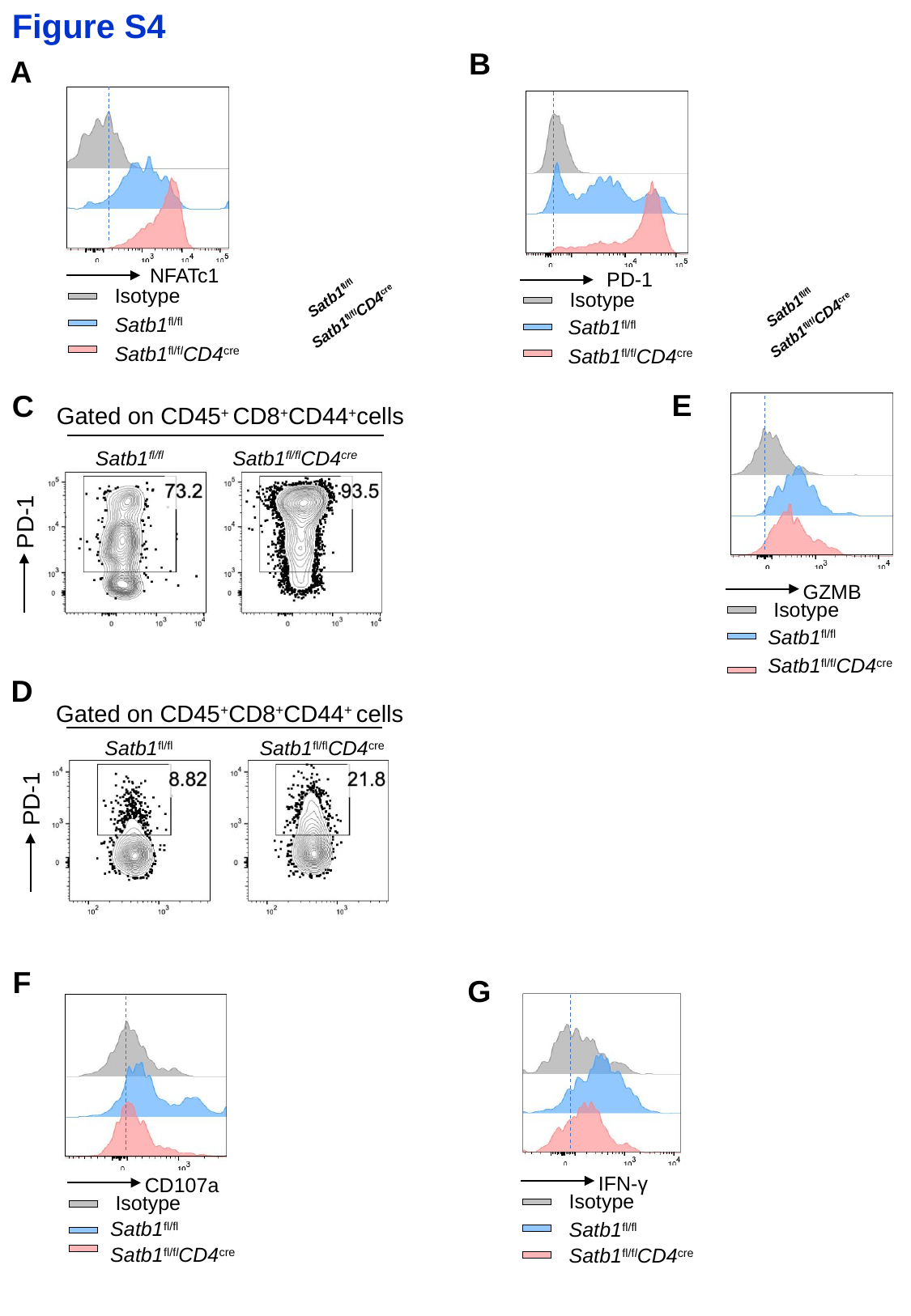

Figure S4
B
A
NFATc1
PD-1
Satb1fl/fl
Satb1fl/fl
Isotype
Isotype
Satb1fl/flCD4cre
Satb1fl/flCD4cre
Satb1fl/fl
Satb1fl/fl
Satb1fl/flCD4cre
Satb1fl/flCD4cre
E
C
Gated on CD45+ CD8+CD44+cells
Satb1fl/fl
Satb1fl/flCD4cre
PD-1
GZMB
Isotype
Satb1fl/fl
Satb1fl/flCD4cre
D
Gated on CD45+CD8+CD44+ cells
Satb1fl/fl
Satb1fl/flCD4cre
PD-1
F
G
IFN-γ
CD107a
Isotype
Isotype
Satb1fl/fl
Satb1fl/fl
Satb1fl/flCD4cre
Satb1fl/flCD4cre

## Slide 5
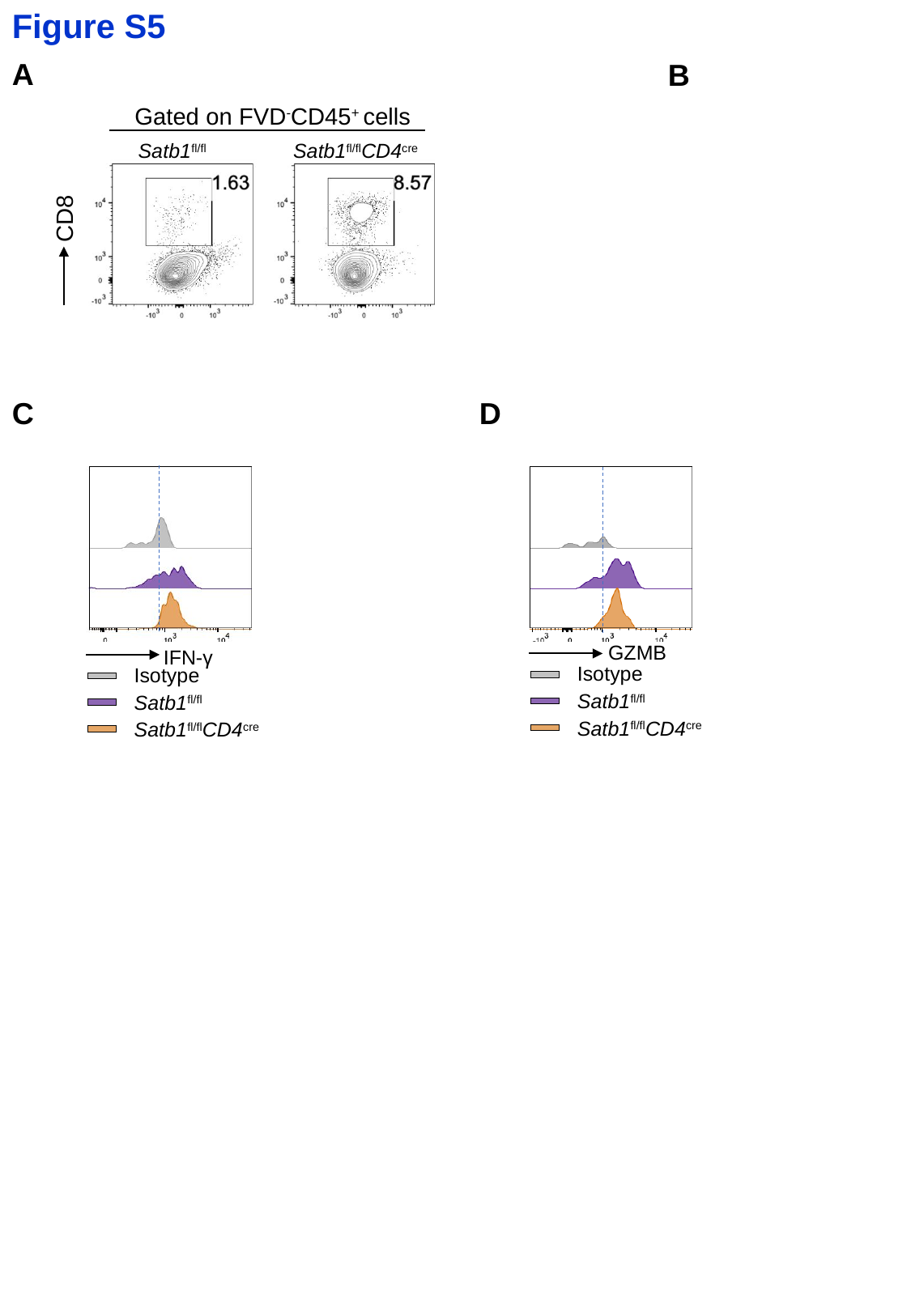

Figure S5
A
B
Gated on FVD-CD45+ cells
Satb1fl/fl
Satb1fl/flCD4cre
CD8
C
D
GZMB
IFN-γ
Isotype
Isotype
Satb1fl/fl
Satb1fl/fl
Satb1fl/flCD4cre
Satb1fl/flCD4cre

## Slide 6
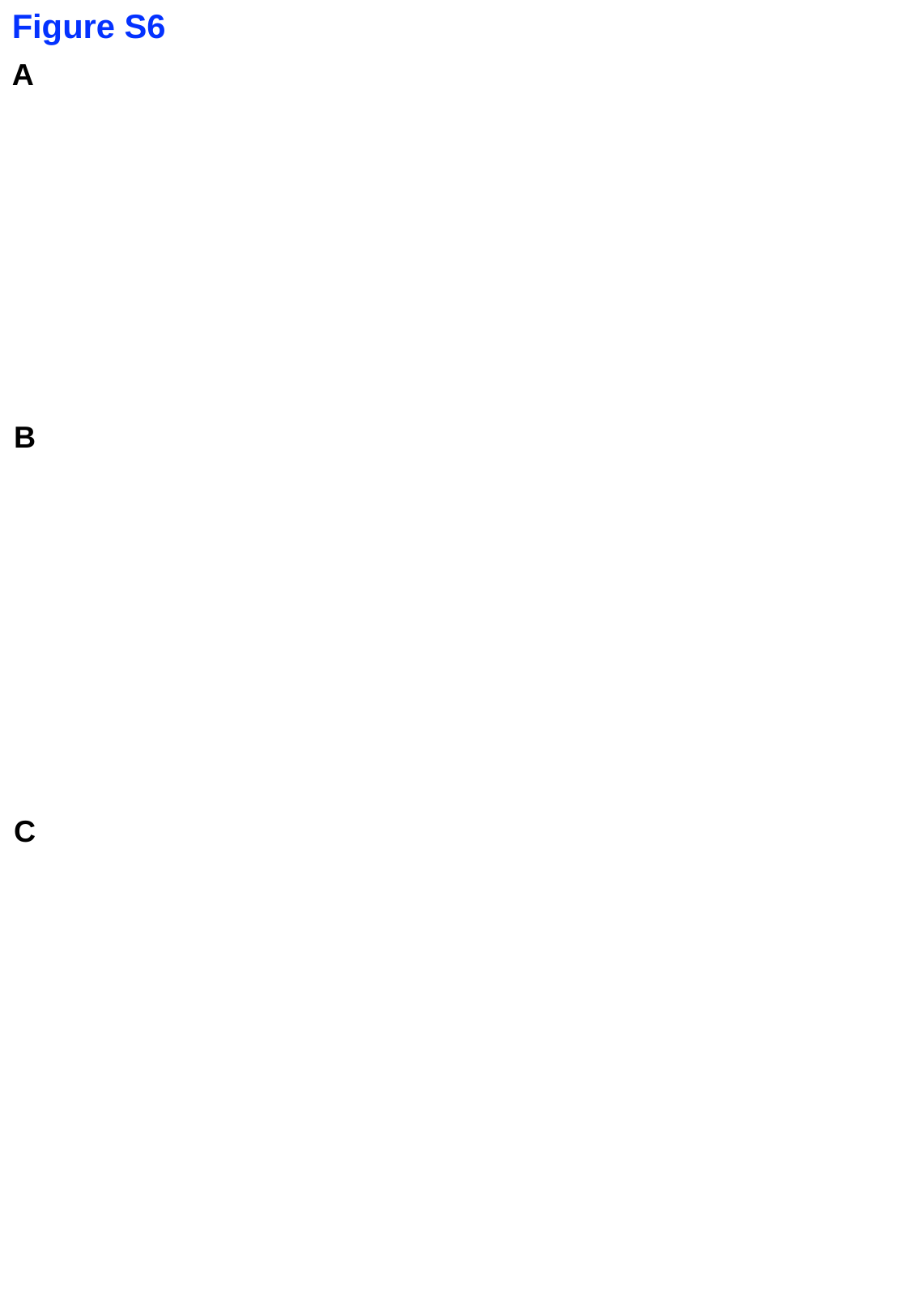

Figure S6
A
B
C
